# Teachers’ roles and identities in student-centered classrooms

**DOI:** 10.1186/s40594-018-0131-6

**Published:** 2018-09-14

**Authors:** Leslie S. Keiler

**Affiliations:** Department of Teacher Education, York College, The City University of New York, 94-20 Guy Brewer Boulevard, Jamaica, New York, USA

**Keywords:** Teacher identity, Teacher roles, Peer leadership, Professional development, Student-centered classes

## Abstract

**Background:**

Students and teachers in twenty-first century STEM classrooms face significant challenges in preparing for post-secondary education, career, and citizenship. Educators have advocated for student-centered instruction as a way to face these challenges, with multiple programs emerging to shape and define such contexts. However, the ways to support teachers as they transition into non-traditional teaching must be developed. The purpose of this study is to explore the impacts on educators of teaching in student-centered, peer-mediated STEM classrooms and preparing student peer leaders for their roles in these classes. Research questions examined how teachers think about themselves as they implement student-centered pedagogy, the difficulties they face as their roles and identities shift, and the ways they grow or resist growth. Qualitative research conducted at two urban secondary schools documents the diverse experiences and responses of teachers in an innovative, student-centered STEM instructional program. The experiences and perceptions of 13 STEM teachers illuminate the possibilities and challenges for teachers in student-centered classrooms.

**Results:**

All participating teachers described multiple benefits of teaching in a student-centered classroom and differences from traditional classrooms. Their transitions to this type of teaching fell into three major categories based upon past identities and current beliefs. Some teachers found the pedagogy consistent with preexisting identities and embraced it without radical change to their concepts of teaching. They described ways in which the model helped them become the teachers they had always wanted to be. Other teachers, who initially identified as deliverers of STEM content, had more difficult experiences adjusting to student-centered instruction. In one case, a teacher resisted change and exited the program, maintaining her identity and deciding not to become student-centered. Other participating teachers made dramatic shifts in their identities in order to implement the program. These teachers described significant learning curves as they shared responsibility for student learning with student leaders.

**Conclusions:**

This study suggests that radically changing the learning environment can affect teachers’ identities and their approaches to teaching in predictable ways that can inform teacher education and professional development programs for STEM teachers, maximizing the success of teachers as they implement student-centered pedagogy.

## Background

Students and teachers in twenty-first century secondary STEM classrooms face significant teaching and learning challenges in preparing for post-secondary education, career, and citizenship. This preparation extends far beyond mastery of content knowledge, which has been the focus of traditional STEM instruction. The Partnership for 21st Century Learning (2015) includes *Learning and Innovation Skills* in its Framework for 21st Century Learning. They define these learning and innovation skills as creativity and innovation, critical thinking and problem solving, communication, and collaboration. The Next Generation Science Standards (NGSS) are consistent with this focus on twenty-first century skills. The argument for why the new standards are important states:

Science—and therefore science education—is central to the lives of all Americans. A high-quality science education means that students will develop an in-depth understanding of content and develop key skills—communication, collaboration, inquiry, problem solving, and flexibility—that will serve them throughout their educational and professional lives (NGSS, Lead States 2013, Why section)

The National Council of the Teachers of Mathematics (n.d.) include in their Six Principles of School Mathematics concepts relevant to twenty-first century skills: 1) Teaching: “Effective mathematics teaching requires understanding what students know and need to learn and then challenging and supporting them to learn it well,” and 2) Learning: “Students must learn mathematics with understanding, actively building new knowledge from experience and previous knowledge” (p.2). The Partnership for 21st Century Learning defines learning environments that will support the development of these critical skills, requiring professional development that will facilitate a significant pedagogical shift.

### Reasons for student-centered STEM instruction

Research about student-centered instruction in STEM, with students taking an active role in the learning process rather than being passive recipients of information from the teacher, demonstrates outcomes consistent with developing 21st century skills and STEM mastery. A variety of instructional models in STEM classes define themselves as student-centered (Boddy et al. 2003; Kazempour 2009; Moustafa et al. 2013; Odom and Bell 2015; Qhobela, 2012; Tamim and Grant 2013; Yukhymenko et al. 2014). Research about such models has tended to focus on the experiences of and outcomes for the students, which are largely positive in both cognitive and affective domains. Educators have used constructivist theory to develop a variety of student-centered instructional approaches, each with its own research base and consistently positive student impacts. Research about student-centered, constructivist classrooms documents increases in students’ higher order thinking, learning, and motivation, particularly in STEM classes (Boddy et al. 2003; Moustafa et al. 2013). Research about specific models highlights commonalities across constructivist, student-centered STEM learning environments. For example, inquiry-based instruction, grounded in constructivist theory, has yielded a variety of benefits for students including learning of STEM content and process skills, increased levels of engagement, positive attitudes about science, and enhanced non-cognitive skills (Juntunen and Aksela 2013; Kazempour 2009; Keys and Bryan 2001; Odom and Bell 2015). Problem-based learning (PBL), another student-centered approach that requires groups of students to explore real-world problems, has been shown consistently to increase performance in science courses and enhance science content knowledge, as well as improve critical thinking, student dispositions, student behavior, and attitudes about learning (Burris and Garton 2007; Gordon et al. 2001). Project-based learning (PjBL), a similar student-centered model that extends solving a problem to completing a project, has been linked to gains in student motivation, critical thinking, and academic skills in STEM classes (Tamim and Grant 2013). Further, research demonstrates that STEM classes that implement democratic science pedagogy support the development of critical science agency among participating urban students, which “opens doors for students to engage in science and to redress power differentials in their lives” (Basu and Barton 2010, p. 86). This student empowerment, which happens in urban STEM classes focused on social justice, enacts Friere’s goal of student agency (Gutstein 2007). This set of examples from specific student-centered pedagogies illustrates a pattern of positive impacts of student-centered instruction in STEM classes. This diverse range of benefits across multiple studies and contexts justifies the increasing pressure on STEM teachers to implement student-centered instruction (Lew 2010), including in current teacher assessment systems (see Danielson 2014).

### Teachers’ roles and responsibilities in student-centered STEM classrooms

Effective implementation of novel pedagogies requires understanding teachers’ roles and responsibilities in the transformed classrooms. The student-centered classroom literature defines the teacher’s roles and responsibilities in classes that employ student-centered pedagogies, including various iterations of constructivist and inquiry-based instruction. According to Moustafa et al. (2013), in constructivist classes “(t)he teachers’ role is to encourage and accept student autonomy and create a comfortable atmosphere for student expression,” acting as guides for their students (p. 418–419). Constructivist teachers behave in marked contrast to traditional instruction where teachers dominate the classroom and provide direct instruction focused on content knowledge acquisition. Friere saw teachers as partners of students who were pursuing agency as opposed to teachers being “positioned as enforcers, disciplinarians, and police officers” (Gutstein 2007, p. 424). Teachers who implement democratic STEM pedagogy must learn to share authority with their students, enabling the student to make instructional decisions that the teachers support and enact (Basu and Barton 2010). Again, specific examples of research in several student-centered instructional models illustrate common themes of impacts of student-centered environments. Researchers describe teachers in inquiry-based classes as catalysts, who act largely through guiding questions (Juntunen and Aksela 2013). According to Yukhymenko et al. (2014), “In a PBL environment, the teacher is not the information provider or classroom controller. Rather, the teacher facilitates, coaches, and models good problem solving skills for their students” (p. 102). Tamim and Grant (2013) identified four roles of teachers in PjBL classes: reinforcer, extender, initiator, and navigator. Thus, across the literature, teachers in different types of student-centered classes take on, or are supposed to take on, the roles of facilitators and instructional managers.

In order to fulfill their new roles, teachers must shift their focus in the classroom from lecturing to assessing. Inquiry-based teachers become assessors because “it can help in diagnosing students’ prior knowledge, gauging students’ understanding throughout the learning experience and guiding instruction, and measuring their understanding and knowledge at the completion of the learning experience” (Kazempour 2009, p. 56). In other words, “(t)he facilitator maintains the focus on learning, guides the process, meters the challenge, and provides appropriate feedback to each student and the whole group” (Gordon et al. 2001, p. 171). This change in focus represents a dramatic shift from past practice.

The literature documents some of the challenges that teachers face when implementing student-centered pedagogies. Identified obstacles to becoming student-centered include concerns about time to cover the curriculum (Boddy et al. 2003; Kazempour 2009; Keys and Bryan 2001; Tamim and Grant 2013), anxieties over students’ performance on external exams (Kazempour 2009; Keys and Bryan 2001; Qhobela, 2012; Tamim and Grant 2013), resistance to change from traditional methods (Qhobela, 2012), peer pressure from other teachers (Lewis 2014), lack of flexibility in the classroom (Tamim and Grant 2013), tendency to teach as they were taught (Kazempour 2009; Lewis 2014), and apprehensions about classroom management (Tamim and Grant 2013). This literature focuses on the reasons teachers provide for resisting implementation, most of which focus on students, rather than what the teachers believed about themselves as they attempt to be student-centered. While there are some reports of teacher stress connected to reform-related curriculum that includes mandates for student-centered instruction, little literature addresses impacts on teachers as they implement student-centered pedagogy (Lewis 2014), the focus of the current study. As the teacher identity literature reviewed below indicates, understanding impacts of this dramatic environmental change on teachers’ identity is critical for understanding and affecting teachers’ instructional decision-making.

### Teacher identity

Beijaard et al. (2004) document the diverse meanings of teacher identity in the literature. For the purposes of this study, *teachers’ roles* refer to what teachers do in classrooms and *teachers’ identities* refer to the ways that teachers think about themselves and their classroom roles. This work builds on Grier and Johnston’s (2009) argument that, “Teacher identity is based upon the core beliefs one has about teaching and being a teacher that are constantly changing and evolving based upon personal and professional experiences” (p. 59). The current study expands the literature by examining how a particular pedagogy affects teachers’ identities as they learn to implement a new instructional model. Reviewing the teacher identity literature, Davis et al. (2006) argue that teachers’ personal histories and professional experiences influence their professional identity development. While teachers’ experiences are central to their identity development, these experiences are processed within a particular context and influenced by a community of practice (Freedman and Applement 2008; van den Berg 2002; van Huizen et al. 2005). According to Basu et al. (2009), “In our use of the term identity, we align ourselves with those who view identity as fluid and constructed socially within communities of practice” (p.360). Proweller and Mitchener (2004) argue that students play a central role in the development of their teachers’ professional identities. In the current study, students who act as peer leaders form a major component of the teachers’ communities of practice, increasing the complexity of the teachers’ professional context. Much of the research on teacher identity development focuses on pre-service teachers (Merseth et al. 2008; van Huizen et al. 2005) and/or early career teachers (Davis et al. 2006) across contexts, while the current study includes teachers across a range of professional experience in a particular instructional environment.

In another perspective on identity, Cohen (2008) argues, “teachers’ identities are central to the beliefs, values, and practices that guide their engagement, commitment, and actions in and out of the classroom” (p. 80). The literature demonstrates that past experiences affect teacher identity, which then modulates their pedagogical choices (Eick and Reed 2002; Rex and Nelson 2004). Thus, experiences shape teachers’ identities (Proweller and Mitchener 2004), and teachers’ identities affect their experiences as their identities influence their instructional practice. The current study uses this framework of evolving teacher identity to investigate the relationships among teachers’ classroom roles and responsibilities, the ways they think of themselves as professionals, and their attitudes and beliefs about their students. It explores the ways that a particular student-centered instructional model affects teachers’ identities and their classroom behaviors.

### Study objectives

The purpose of this study is to explore the impacts on urban STEM teachers of preparing peer leaders for their roles in student-centered, peer-mediated classrooms and teaching classes through these peer leaders. Research questions examine how the teachers view their professional roles and identities as they participate in student-centered classes, the challenges they face as roles and identities shift, and the ways they grow or resist growth. The study explores these issues within the *Peer Enabled Restructured Classroom* (PERC) Program, in which typical high school students act as peer leaders in STEM classes, facilitating instruction on a daily basis. The PERC Program was developed to improve performance in STEM classes, increase high school graduation rates, and expand college readiness for non-honors students in urban schools. The ways in which teachers respond to the PERC instructional model contribute to understanding how to shift classroom practice toward student-centered instruction across multiple contexts. Thus, the research questions that guided this study were:

- How do teachers think about themselves as they implement student-centered instruction?
- What rewards and challenges do teachers experience as their roles and identities shift in student-centered classrooms?

## Methods

### Research design

Case studies (Yin 2014) of a program that implemented the student-centered PERC instructional model at two secondary schools were developed in order to answer the research questions, examining the diverse impacts of teaching in this context on teachers’ conceptions of their roles and identities. All 13 teachers who participated in the PERC program at the two schools were included in the case samples, in addition to their administrators, mentors, and coaches. Students were not considered participants because the data was not collected from them directly, although they were observed during PERC classes. Two schools were selected for exploration because, as Miles et al. (2014) argue, “multiple cases offer the researcher an even deeper understanding of the processes and outcomes of cases” (p. 30), although two cases still does not enable generalizability. The two current case studies used qualitative methods (Huberman and Miles 1994; Merriam 2009; Miles et al. 2014; Stake 2010) to inquire into issues of teacher identity and development. As Merriam (2009) argues, qualitative methods are appropriate when research questions focus on “(1) how people interpret their experiences, (2) how they construct their worlds, and (3) what meaning they attribute to their experiences” (p. 23). These characteristics were critical to the design of the current study. The case studies were *descriptive explanatory* in nature, seeking to describe and explain patterns related to the phenomena and relationships that influence the phenomena (McMillan and Schumacher 2006). The case studies are situated within a larger study of the diverse impacts of the PERC Program (see the “Study Site and Participants” section below) on schools, students, and teachers that is designed to make contributions to practice (McMillan and Schumacher 2006). The researcher acted as a *participant observer* (Creswell 2002, 2013), as the PERC Director of Teacher Development, leading the professional development (PD) team and providing coaching to some of the teachers. This role provided deep and extended access to the study participants (Creswell 2002, 2013). However, it did necessitate steps to avoid bias (Yin 2014) that result from researcher effects. Miles et al. (2014) suggest that researchers take steps to minimize bias such as including extensive time at the site, including participants with diverse views and experiences, checking emerging patterns with participants, and triangulation of data (McMillan and Schumacher 2006; Stake 2010). The researcher and PD team spent extensive time at the case schools. The three PERC coaches were at the case schools on a weekly basis collecting data as they supported the teachers’ development. The researcher visited the new school every week and the mature school once per month for a full school day, providing feedback to the teachers and coaches while working to increase fidelity of implementation across schools. The entire PERC PD team, including the Director of Teacher Development, the three PERC coaches, the TAS Pipeline Coordinator, and the Sustainability and Growth Coordinator, discussed the coaches’ observations and analyses, offering insights from multiple perspectives and experiences. This study included all teachers involved in PERC in the case schools to avoid selection bias. Preliminary study findings were presented to PERC teachers with various levels of experience in the program, coaches, TAS, and researchers working on different topics during a Summer Institute research presentation. The audience was asked to provide written and oral feedback about the findings, which were revised in response to suggestions.

### Procedures

The study incorporated a variety of data sources in order to facilitate triangulation (McMillan and Schumacher 2006; Stake 2010). The study focused on teacher interviews and focus groups in order to prioritize teachers’ perspectives about their experiences. Other data sources were used to contextualize and enrich the interpretations of the teachers’ claims and explanations. Teachers, administrators, and coaches participated in semi-structured interviews (Creswell 2002, 2013; Kvale, 1996) and focus groups (Creswell 2002, 2013; Krueger and Casey 2009) that enabled them to describe their own experiences and perspectives while staying focused on the context of the program under study. Interviews and focus groups were conversational in nature and included “probing questions to clarify and refine the information and interpretation” (Stake 2010, p. 95). Data collection focused on the 13 mathematics and science teachers with experience in the PERC Program in these two schools. Individual interviews and focus groups were conducted during June before teachers began PD in the PERC Summer Institute, during August at the end of the Institute, and during October and May of each year of program participation. Interviews and focus groups lasted from 20 to 90 min, depending upon the teacher’s schedule and length of responses. Interviews and focus groups were recorded and transcribed. Program coaches observed teachers every week during their first year in PERC and either every week or every other week during teachers’ second year in the program, depending upon perceived needs for support. The coaches documented their observation with field notes using *low inference descriptors*, notes that describe events without evaluation (McMillan and Schumacher 2006), and an observation protocol completed by program coaches based upon PERC class target behaviors (see Appendix 1). The Director of Teacher Development trained each of the three coaches on how to take low inference notes and complete the observation protocol. Training involved joint observations by the Director and multiple coaches, who then compared and analyzed their documentation. Training continued until coaches reached 100% agreement on three joint observations, with monthly checks between the coaches and the Director to maintain levels of agreement. The PD team used this documentation to monitor fidelity of implementation of the model and establish PD goals for individual teachers and the program as a whole. Coaches uploaded completed observation reports into the PD team database and shared them with the teachers themselves. Coaches discussed teacher progress and challenges at half-day PD team meetings that occurred every other week, making plans for individual and collective teacher support. Data was collected across three years (2013–2014, 2014–2015, 2015–2016) in the mature PERC school and for the one year (2015–2016) of program participation in the school that was new to PERC.

### Data analysis

Following Yin (2014), Merriam (2009), and Miles et al. (2014), the various data sets were analyzed and interpreted to develop findings, themes, and patterns. Pattern development was an iterative process, with codes emerging from the interview and focus group transcripts and then contextualized by the other data sets. The researcher analyzed the full range of data sets after each round of interviews adding to and modifying codes over the three years of the study. One PERC PD team meeting each semester was devoted to reviewing the preliminary findings in order to inform both the data analysis and the planning of teacher PD. The three coaches, the TAS coordinator, and the Sustainability and Growth coordinator provided verbal feedback about the consistency of the emerging patterns and findings with their experiences in the field. At the end of each semester, the PERC teachers were asked to confirm or refute preliminary findings, which were then modified based upon their responses. Thus, while a single researcher conducted the analysis of the raw data, the patterns and findings were reviewed and verified by multiple program participants.

### Study site and participants

#### PERC class

In the PERC Program, teachers and students collaborate to implement a novel instructional model that leads to improved STEM learning and performance (Thomas et al. 2015) as well as increased college readiness (Bonner and Keiler 2015) for students in high needs urban schools. A typical PERC lesson begins with whole class instruction and ends with individual formative assessment, but the majority of class time every day is spent with students working in small groups of approximately four students led by a peer instructor and supervised by the teacher. The PERC class structure is consistent with examples of student-centered instruction in the literature reviewed above. The peer instructors, called Teaching Assistant Scholars (TAS), are students who passed the STEM course and perhaps the associated high stakes exam the year before at a level that qualified them for high school graduation but not for college success. Teachers select TAS for the following year from their current students who have patterns of attendance and positive peer interactions that PERC has discovered leads to being an effective TAS. During the PERC lesson, the teacher, seven to eight TAS, and approximately 30 students engage in PERC class target behaviors (Appendix 1) that support the learning and success of everyone in the classroom. TAS are responsible for ensuring that students are on task and learning throughout the lesson. TAS monitor their students’ developing mastery of the material, ask scaffolding questions, provide feedback, and serve as role models for appropriate academic engagement. In PERC class, teacher target behaviors largely involve guiding the work of the TAS and assessing TAS performance and student learning. Whole class, teacher-centered instruction is limited to 20% of class time. This classroom structure varies dramatically from traditional STEM classes, and teachers’ responsibilities within a PERC classroom diverge considerably from what they do in non-PERC classes.

#### TAS class

In addition to their PERC classes, PERC teachers teach a course specifically designed for their TAS, which is part of the teachers’ regular instructional load and the TASs’ normal course schedule. The TAS class curriculum is divided into four components: learning to teach, learning to learn, learning content, and college knowledge. All components of the course are research-based. In learning to teach lessons, PERC teachers help their TAS learn how to support their students’ learning in PERC class through activities such as asking scaffolding questions, conducting formative assessment, and providing targeted feedback. Such tutoring-type experiences have been shown to have positive impacts on the peer leaders’ academic performance and self-concept (Ginsburg-Block et al. 2006; Komives et al. 2006; McMaster et al. 2006; Morrison 2004; Robinson et al. 2005; Roscoe and Chi 2008; Topping 2005). In learning to learn lessons, the PERC teachers work to develop the TASs’ own academic skills and self-regulation, focusing on goal setting, time management, and metacognition, which have been linked to college-ready skills and attitudes (Dignath and Büttner 2008; Greene and Azevedo 2007; Kistner et al. 2010; Perels et al. 2005; Schunk and Ertmer, 2000; Zimmerman 2008). Learning content lessons supplement the TASs’ incomplete mastery of the content developed the prior year and include advanced subject area exploration. Finally, in college knowledge lessons, the teachers guide the TASs’ investigation of what it takes to be admitted to and succeed in college and careers. Such college and career awareness has been linked to college success, especially for students in under-resourced urban schools (Cates and Schaefle 2011; McDonough 1997; Stanton-Salazar 2001; Tai et al. 2006). Of the four components, only learning content lessons in any way replicates the teachers’ prior experience and responsibilities. Across the course, teachers work with TAS to develop Conley’s (2008) academic knowledge and skills essential for college readiness. Thus, the TAS class requires the PERC teachers to engage in a completely novel instructional experience as they support their TASs’ development.

#### PERC student impacts

The PERC Program has demonstrated a range of impacts on participating students (Bonner et al. 2017; Bonner and Thomas 2017; Gerena and Keiler 2012; Keiler 2011b; Thomas et al. 2015). TAS are much more likely to exceed passing scores and meet proficiency standards for college readiness after participating as peer leaders. For example, in 2013–2014, 47% of students reached the state college readiness benchmark after being a TAS as compared to 15% before this experience, with 14% achieving the state defined Mastery level post-participation compared to 1% pre-participation (CASE 2015). Further, preliminary analysis of TAS high school graduates suggests that about 73% of them enrolled in post-secondary education, far above the New York City average (CASE 2015). PERC students have also demonstrated positive outcomes on state examinations. For example, in the 2014–2015 cohort, Algebra I students were 1.6 times more likely to pass the Regents exam as matched peers in peer high schools (CASE 2015).

#### PERC Program professional development

In order to support teachers as they learn to implement the PERC and TAS classes, the PERC Program created a PD model using research-based best practices (Keiler 2011a; Keiler and Robbins In Press; Darling-Hammond and Richardson 2009; Ermeling and Gallimore 2014/2015; Gusky 2000; Gusky and Yoon 2009; Showers and Joyce 1996; Topping 2001; Tschannen-Moran and Tschannen-Moran 2011). The purpose of the PERC Professional Development program was to create a PERC community of practice within schools and across schools that, through teacher collaboration and inquiry, becomes well-versed in PERC. It was teacher-need driven and embedded in the daily work of participating teachers, fulfilling Gusky and Yoon’s (2009) concept of “just-in-time” PD. Since different forms of PD offer different strengths and are subject to different shortcomings (Gusky 2000), the PD model include a variety of experiences described below. Teachers who entered the PERC Program, regardless of how long they had taught previously, were referred to as *Novice PERC Teachers*. Novices participated in a 2-year PD program (see Appendix 2) once they joined PERC. The PERC Program has scaled in two ways, expanding to new schools and expanding within schools. Some schools, like the mature school in this study, started with a single subject area, adding teachers and subject areas each year until they reach capacity for teachers who want to participate. Other schools, like the study school in its first year in PERC, started with multiple teachers and subjects. In either instance, schools replace teachers who leave the school or the program to pursue other opportunities. Thus, PERC schools may have had teachers at different phases of the program, depending upon when each teacher began the program.

Teachers moved through the PD model in distinct phases: (1) introduction to the PERC Program, (2) PERC Program induction during the Summer Institute, and (3) academic year PD including school-based coaching and PD workshops. Teachers’ introduction to the PERC Program involved visits to PERC schools, where they observed classes and interacted with experienced PERC teachers and TAS. They were able to ask questions of PERC teachers and students, administrators, and PERC staff. These visits formed the basis of teachers’ decisions about whether to enter the PERC Program. These visits are essential as teachers are more willing to implement pedagogies they have observed in actual classrooms (Topping 2001). During the summer before their first academic year in the program, novice PERC teachers were immersed in the model during the 6-week, 4-days-per-week PERC Summer Institute. The PERC Summer Institute served as a PD lab for the program while providing remedial coursework for students who failed state exams in June and needed to retake the exams in August. Each day began with 1 h of TAS class, followed by 4 h of PERC class. Novice PERC Teachers’ summer experiences included (1) being mentored by a lead PERC teacher in a PERC class, (2) working with TAS summer interns to implement the class, and (3) being coached by the PERC PD team during and outside the PERC class. Additionally, during afternoon 2-h PD workshops, the novice PERC teachers learned TAS class routines and were introduced to the TAS class curriculum.

During their first year in the program, teachers received weekly school-based coaching that included observations of their PERC and TAS classes and hour-long coaching sessions that focused on teacher reflection and goal setting. During their second year, school-based coaching was either weekly or every other week, depending upon the PD team’s assessment of the teacher’s needs. During their third year and thereafter, PERC coaches visited each teacher once per month to continue to provide support and monitor fidelity of implementation of the model. PERC teachers formed a community of practice at their schools and joined a PERC teacher community across PERC schools. PERC teachers came together for monthly all-day workshops that enabled them to share successes and collaborate on meeting challenges. Additionally, teaching the TAS class and mentoring the TAS were PD experiences in and of themselves, as teachers developed as reflective practitioners when they taught pedagogy to and assessed the performance of their TAS. The PD team referred to teaching the TAS class as *stealth PD* because the coaches were able to shift teachers’ practice by having them teach desired pedagogies to their TAS, which led to the teachers implementing and reflecting upon the practices themselves. Once they developed mastery of the PERC and TAS classes, teachers could become mentors to new PERC teachers, further developing their skills in implementing the model as they grew as reflective practitioners.

#### Participants

All 13 teachers in the study (see Table 1) were certified in their field of instruction. They ranged in teaching experience from second year teachers to veterans of 13 years in the classroom when joining PERC. Eleven of the teachers were white and two black, all non-Hispanic, and four were female and nine male. Pseudonyms are used for all teachers. The 13 participating teachers had varying years of experience and levels of involvement in the PERC Program during the study. Nine of the participating teachers (Bill, Hillary, Jerry, Paula, Henry, Matthew, Alice, Andrew, and Lily) joined PERC and experienced PERC PD within the timeframe of the study. Their insights focused on this recent transition to PERC teaching. Two teachers at the mature school (Alan and Peter) were PERC teachers in the earliest years of the program, but had moved into other responsibilities within their schools and taught classes not in the PERC model during the time of the study. They were able to reflect upon their experiences becoming PERC teachers, their observations of the teachers in their school who replaced them in PERC classes, and contrast their PERC and non-PERC teaching experiences. One teacher (Mark) at the mature school experienced the PERC Program orientation, involving visiting PERC classes and doing a needs assessment with PERC coaches, in preparation for the following year. Mark contributed his predictions about what would be challenging for him and his insights about observations of PERC teachers in his school. One teacher (Bob) did not elect to become a PERC teacher himself, but teamed with a Special Education teacher (Henry) who did become a PERC teacher. Bob was able to compare his PERC and non-PERC classes and his role within those, even though he did not participate in PERC PD himself. These diverse experiences resulted in different quantities of contributions to difference aspects of the data sets and results discussed below.

**Table 1:** Professional experience and teaching assignments of 13 participating teachers at the two case study schools

		Mathematics				Science		
Mature school	Pseudonym	Alan	Bill	Hillary	Peter	Jerry	Paula	Mark
	Certification	Math 7–12	Math 7–12	Math 7–12	Math 7–12	Physics 7–12	Chemistry 7–12	Earth Science 7–12
	Teaching assignments	4- Algebra II (11th grade) (Former PERC Algebra)	2- PERC Algebra (9th grade), 2- non-PERC Algebra, 1- TAS class	2- PERC Algebra 9th grade), 2-non-PERC Algebra, 1- TAS class (when in PERC)	Math dept. mentor, 3- Non- PERC Geometry (Former PERC Algebra)	2- PERC Physics (11th and 12th grade), 1- Non-PERC Physics, 1- TAS Class	2- PERC Chemistry (10th and 11th grade), 1- AP Chemistry, 1- TAS Class	3- Non-PERC Earth Science (future PERC Earth Science), 2- Exploring Computer Systems
	Experience level	Veteran	2nd year	Veteran	Veteran	3rd year	Veteran	Veteran
	Type of transition	Difficult	Easy	None	Difficult	Easy	Easy	Difficult
New school	Pseudonym	Bob	Henry	Matthew		Alice	Andrew	Lily
	Certification	Math 7–12	Special education	Math 7–12		Special education	Chemistry 7–12	Biology 7–12
	Teaching assignments	1- PERC Geometry (10th–12th grade) with Henry (content teacher, no PERC PD), 3- Non-PERC Geometry	1- PERC Geometry (10th–12th grade), 1- Non-PERC Geometry, 1- Non-PERC Algebra, 1- TAS class	2- PERC Algebra (9th grade), 2- non-PERC Algebra, 1- TAS class		1- PERC Chemistry (11th and 12th grade) with Andrew, 3- non-PERC Art, 1- TAS class	3-PERC Chemistry (11th and 12th grade), 1- non-PERC Chemistry, 1- TAS class	3-PERC Living Environment (9th grade), 1-Non-PERC Living Environment ESL, 1- TAS class
	Experience level	Veteran	Veteran	Veteran		Veteran	Veteran	2nd year
	Type of transition	Difficult	Easy	Easy		Easy	Difficult	Easy

Veteran = tenured and at least 5 years of teaching experience

The two study schools served students with demographics typical of the range of non-selective New York City public high schools (Table 2). In both schools, attendance rates hovered around 80%. The majority of students at both schools came from populations underrepresented in STEM, whether through ethnicity, family income, home language, learning needs, or some combination of these factors. Across the program, students in PERC classes were unlikely to have scored at the proficient level or above on their 8th grade Mathematics or English/Language Arts (ELA) state assessments, with fewer than 30% achieving this standard on the math exam and fewer than 20% on the ELA. Only 10% of entering PERC students had achieved proficient or above on both their 8th grade math and ELA exams. TAS had similar pre-PERC performance data, with less than 55% of them scoring at proficient in math and less than 20% on ELA in the 8th grade (Bonner and Keiler 2015). These characteristics qualified the schools for participation in the PERC Program, which targeted high needs schools that had struggled to meet the needs of students underrepresented in STEM.

**Table 2:** The two study schools that served students with demographics typical of the range of non-selective New York City public high schools

	Asian (%)	Black (%)	Hispanic (%)	White (%)	Special needs (%)	ELLs (%)	Free lunch (%)	HS grad (%)	College ready (%)	Post-secondary enrollment (%)
Mature school	2	26	71	1	28	10	77	75	10	42
New school	1	14	82	3	28	25	80	< 60	18	35

Demographic data is for the 2013–2014 school year and student performance data is for the year prior to joining PERC. “College Ready” refers to NYC DOE standards (https://www.schools.nyc.gov/school-life/learning/college-and-career-planning/college-and-career-glossary)

## Results

All 13 participating teachers discussed ways in which being a PERC teacher differed from their previous teaching or their current teaching in non-PERC settings. The roles and identities that were special to teachers in PERC classes were common across participants. Similarly, the benefits to role shifts that teachers identified in PERC classes were shared among participating teachers. What differed among the teachers was how challenged they were by making the transition to the PERC model. Within this group of 13 teachers, seven (Bill, Jerry, Paula, Henry, Matthew, Alice, and Lily) described an easy transition, five (Alan, Peter, Mark, Bob, Andrew) described a difficult transition, and one (Hillary) did not transition at all, dropping out of the program after one year. Each of these teachers identified aspects of their experiences and identities that facilitated and/or impeded the transition. Similar patterns in teacher experiences appeared in both case study schools, with no observable differences appearing across sites. Thus, the results are reported together. Typical quotes from the themes that appeared in the data are used to illustrate each theme.

### PERC teacher roles and identities

All PERC teachers in the study described experiencing changes in the roles they played in the classroom and shifts in their identities as teachers that accompanied these role changes. A major alteration involved the teachers’ roles and identities concerning content. Matthew, a veteran Algebra teacher, contrasted his roles in the PERC class with his non-PERC classes:

My role in PERC class is completely focused on how each group is progressing as far as working with their TAS and kind of randomly doing small observations of my TAS and trying to make sure that the kids are being challenged appropriately. Whereas in a non-PERC class I am just focused on content and making sure all my students are actually learning math from *me*. So it’s a completely different experience.

In Matthew’s PERC class, he focused on the students, whereas in his other classes, the students focused on him. In PERC, he assessed the students’ interactions with content, while in non-PERC, he delivered content himself. Similarly, in the PERC classroom, Andrew, a veteran Chemistry teacher, saw himself in a PERC class as, “Facilitator. A monitor. Also a big default go to, it’s even good to see the TAS sometimes, if they do make mistakes they’ll readily own up to it and they’ll say “OK,” and they’ll ask me over to be a corrector of sorts. And I am also there as an encourager.” All of Andrew’s descriptors focus on his interactions with the TAS and how he taught the PERC students through the TAS, acting as their manager and guide. While he retained the role of content expert in the classroom, his new identities mediated the ways in which he implemented that role, becoming a content resource rather than a content dispenser. Alan, a veteran math teacher, mirrored Andrew and Matthew’s prioritizing of students as he described refocusing his planning after becoming a PERC teacher: “you become aware of how much time you used to be spending worrying about what you are going to say rather than what the students are understanding.” This shift from focus on content to focus on students demonstrated a major change in the teachers’ thinking about themselves as educators.

PERC teachers had individual journeys through these identity transformations, but patterns exist across these journeys. For some teachers, participating in the PERC Program enabled them to embody their desired professional identities, resulting in a smooth and fulfilling transition within the classroom. However, some teachers’ pre-existing identities clashed with the ethos and structure of the PERC classroom. In the majority of these cases, the teachers underwent significant identity transformations as they embraced their new roles in the PERC and TAS classes. In contrast, one teacher’s identity remained intractably in conflict with PERC, causing stress for her and her students in PERC and TAS classes, as well as her coach. While this was less than 10% of the teachers who entered PERC, it poses an important challenge to program implementation and scale.

### Embracing roles and identities

#### Being student-centered

For many PERC Program participants, the experience was, as Matthew described, “a dream come true for a teacher.” Some of the teachers in the study had been waiting their whole careers to fulfill the roles they found in PERC, arguing that the PERC classroom enabled them to be the teachers they wanted to be. For example, Bill’s PERC coach described him as “a natural” because the PERC instructional model seemed to fit so seamlessly into his teacher identity, even as a second year Algebra teacher. Similarly, Bill’s school-based mentor described him as eager to learn and grow in the PERC Program because he valued the roles that it allowed him to play in the classroom. These easy adopters tended to be teachers who had wanted to implement groupwork in their classrooms, but they had previously been unsuccessful in getting students to remain productive while working in groups and usually reverted to teacher-centered instruction. Paula, a veteran Chemistry teacher, argued that she had always favored cooperative learning but that the typical classrooms behaviors of students she taught made implementation unrealistic. As a PERC teacher, supported by a team of TAS, Paula claimed that she was able to implement the kinds of lessons she had always desired.

#### Knowing what students are doing

Some teachers discovered additional, unanticipated benefits to implementing PERC. As Matthew explained, “I have a natural tendency to have students working in groups and have responsibility put on the students to focus and stay on task. So I feel like implementing this program is simple for me.” In non-PERC classes, he continued to ask students to work in groups but was never sure whether they were on task and learning when he stepped from one group to another, reducing his feelings of efficacy as a teacher. In particular, he worried about groups composed of English Language Learners who spoke their native language during class. When he did not speak the students’ language, he did not know if they were discussing math or their weekend plans. PERC made Matthew successful in his preferred instructional modality, increasing his feelings of self-efficacy as a teacher. He described the satisfaction he felt knowing that groups were discussing math with their TAS, regardless of the language they were speaking. Teachers such as these readily adopted the roles necessary to share responsibility for student learning with their TAS and felt fulfilled by their success within these roles. Such teachers experienced satisfaction rather than stress during these role shifts. They had wanted and waited to live the identity of facilitator of learning rather than fount of content.

#### Supporting individual students

In connection with the student-centered nature of the PERC model, PERC teachers described the ways in which the PERC Program enhanced their role of meeting the needs of individual students. While praising the program to potential PERC teachers, Matthew described the insider information that his TAS provided as part of their class discussions about grouping students to maximize success and minimize conflicts. Supporting this perspective, TAS claimed that it was easier for students to be vulnerable and share sensitive information with a peer who could then advocate for them with their teacher. As a second year Biology teacher, Lily argued that because the TAS were keeping all students engaged in PERC class, she was able to sit with groups, have in depth conversations, and support struggling students for extended periods of time. She believed that her role in the PERC class was to deepen the learning experience for individual students rather than ensure that all students were in some way on task. Through their work in PERC classes, participating teachers transitioned from an identity of a teacher of a class to an identity of a teacher of unique students.

In particular, special education teachers quickly embraced the PERC instructional model, while still experiencing significant role shifts. The two special education teachers in the study both taught at the new PERC school, with assignments including partnering with a content teacher in STEM classe into which students with special needs were mainstreamed. Henry was certified in special education and partnered with several different math teachers during his instructional day, including teaching one PERC Geometry class with Bob and one TAS class on his own. Alice was certified in special education and art, teaching several art classes on her own and one PERC Chemistry and one TAS class with Andrew. Employing his expertise as a special education teacher, Henry argued that the student-centered pedagogies of the PERC classroom were an excellent match for their target population, claiming that special education teachers had been attempting to get their general education partners to adopt such approaches for years. However, the two special education teachers also talked about the fact that the TAS were fulfilling many of the student support roles that they played in other classrooms. While this initially led them to question their place in the classroom, they soon realized that the TAS enabled them to employ their expertise in deeper ways. Alice, a veteran special education teacher, explained,

I guess it’s interesting what it does to the role of special education teacher because a lot of times my role when the gen[eral] ed[ucation] teacher is teaching content is conferencing or going around or doing behavior management, making sure kids are on task. All those sorts of things that TAS eliminate, so it actually really allows me to see what students are grasping things and work with them one on one or create strategies on the spot like regrouping or saying something differently or focusing on vocabulary.

Alice, like her colleagues, recognized the vital role she played as an assessor in a student-centered classroom. Eliminating subject expert as the primary role of teachers in a classroom also relieved pressure from teachers who preferred to focus on student learning instead of content. These special education teachers regularly changed subject partners from year to year, needing to master new content annually. The classroom culture of a community of learners matched the special education teachers’ identities of content learner as well, enabling them to feel more successful and useful in their PERC classes than in traditional classrooms. For some special education teachers, the structure of the PERC classroom and the emphasis on their expertise concerning student learning dramatically improved their relationships with their general education teacher partners. Henry, a veteran Special Education teacher, expressed frustration with content specialist partners who lectured the whole class, leaving no room for him as a learning specialist or pedagogies that he knew would be more effective with his population. Administrators and school-based coaches shared the improvements they observed in classroom dynamics and resulting learning opportunities for students of previously contentious teacher pairs. All PERC special education teachers described a true partnership role for them in the PERC classroom, which many of them had not experienced in many traditional classrooms where lecture dominated instruction. They believed that the changing pedagogies and values of the PERC classroom created positive identity shifts for all involved, students and teachers alike.

#### Teacher evaluations

PERC teachers talked about having been pushed by their administrators to implement student-centered classrooms, particularly because of the new evaluation system based upon the Danielson Framework (Danielson 2014). Although Alice claimed she had always had a student-centered approach to teaching as a special education teacher, she articulated the ways that implementing the model would make all teachers successful, “In terms of PERC it’s totally set up for a successful observation. It’s set up, it just looks right in terms of student-centered learning and interactions and all of that it’s totally where education needs to be.” Administrators shared how impressed they were with the dramatic improvement in observation ratings that their PERC teachers received. They claimed that they had urged their teachers to be more student-centered before with minimal results. Andrew, describing prior frustration, explained that he had never known how to make this work and that the PERC Program enabled him to embrace the identity of a highly effective teacher using current educational definitions. Similar teachers recognized the role shifts required by their changing profession and appreciated that the PERC model facilitated growth into a new identity.

#### Relinquishing undesirable roles

Another benefit PERC teachers described was that they got to relinquish roles they did not enjoy, as the TAS either adopted those roles or the presence of the TAS in the groups eliminated the role within the classroom. PERC teachers like Jerry, a third year Physics teacher, claimed that they were relieved to abandon their disciplinarian roles, as “classroom management problems disappeared.” He believed that the TASs’ ability to answer questions immediately and quickly refocus students’ attention on the learning task eliminated the need for a disciplinarian in his PERC classes. Lily, while acting as a mentor, explained to a novice PERC teacher that students in PERC classes were not bored because they got their questions answered immediately by their TAS. Bob, a veteran Geometry teacher, was only involved in PERC through his partnership with Henry, a special education PERC teacher. Bob contrasted his PERC and non-PERC classes, raving about what he was able to accomplish in his PERC classes and how much he struggled on his own in his traditional class, largely because of time on task facilitated by TAS. Alice described the reduction in dealing with behavioral issues that was usually a common role for her as a special education teacher, explaining, “I feel like I put out fires in lot of classrooms that I don’t have to in PERC.” The teachers described here seemed relieved that they were not having to focus time and attention on student behavior in their PERC classes at the expense of supporting student learning, which was common in their non-PERC classrooms.

PERC teachers also argued that the TAS removed roles related to simple explanations of content and procedures. For example, Paula articulated the value of the TAS in the classroom and the shift that it enabled in her role, claiming it was “good to have people take on additional responsibility for the small questions so I can focus on bigger misunderstanding.” She appreciated being able to use her content expertise in complex ways rather than spend time assisting students with basic content facts. Mark, a potential novice PERC teacher in the mature PERC school, predicted that the PERC model would reduce the time he spent repeating instructions to each student and enable him to have genuine conversations about the content he loved. As a veteran Earth Science teacher, he looked forward to shifting classroom roles by joining PERC. The elimination of undesirable, largely management roles enabled the teachers to take on more instructional roles in the PERC classroom, supporting their identity as a professional educator rather than a source of instructions and low-level content, and/or a disciplinarian. In fact, Lily claimed that she had progressed much farther in the curriculum during her first year in PERC than in the previous year because of being able to focus on content. Teachers spoke with enthusiasm about their work in PERC classes in contrast to frustrations they expressed about teacher-centered classes in which they constantly repeated themselves, never getting beyond rudimentary instructions or basic content.

A range of factors contributed to certain teachers making an easy transition to teachers’ roles and identities of the PERC classroom. Easy-transition teachers had pre-PERC identities that were consistent with a collaborative classroom culture in which TAS were trusted to share roles common to teachers in traditional classrooms. They did not value being disciplinarians and focusing on the minutia of content and task instructions. They wanted different relationships with their students and to spend time engaging with meaningful content understanding. Thus, easy-transition teachers felt fulfilled by their roles in the PERC classroom and had identities that led readily to success in the implementation of PERC.

### Resisting the transition to student-centered instruction

While most teachers involved in the program ultimately embraced and succeeded with PERC, one teacher in the mature school was never able to adopt the model. Her identity was antithetical to the model and she remained resistant to change. Hillary, a veteran Algebra teacher, began PD in the PERC Summer Institute claiming that she had always been successful with having students sit in rows and she did not see why she should do anything differently, describing herself as “old school.” When asked what she meant by “successful,” she did not have an answer. During the summer, Hillary focused on tutoring individual students rather than learning how to collaborate with the TAS and implement the model with the entire class. She continued to hold onto her role as content expert and resisted becoming a facilitator and learning team manager. Her observation records demonstrate that most of her instruction remained teacher-centered, with little work being done in groups led by TAS. Few PERC target behaviors were highlighted, especially in the teacher column. Hillary’s coach reported that her TAS classes focused on re-teaching the TAS content rather than mentoring the TAS to develop the leadership and instructional skills they needed to implement the model with their groups. When the program did not work in her classroom during the academic year, she blamed the TAS, saying that she had not had a choice about which students were selected. Both her PERC coach and school-based mentor claimed that Hillary was unwilling to own any of the implementation problems or fully invest in making changes to the way she related to her TAS. Peter, her school-based mentor who was a veteran math teacher and a former PERC teacher, believed that Hillary ultimately did not believe in students’ ability to learn and grow. Peter had experienced a challenging transition himself, working to overcome his affinity for explaining mathematics in order to give students space to master the content with their TAS. Ultimately, according to his coach, Peter’s belief in students facilitated his transformation from a content-deliverer to a facilitator of an instructional team. Hillary’s journey through the PERC Program was complex. While she reported that she had initially been skeptical about the basis of the program—“students teaching students”—Hillary claimed that the program had “won her over” when she saw TAS from her school performing at a high academic level and fulfilling leadership roles in the PERC Summer Institute. However, while she was able to see these benefits to experienced TAS, she never embraced mentoring the TAS and developing their skills and expertise as part of her role as a teacher. Still, Hillary expressed gratitude for her PERC participation, claiming that she had become a better teacher because of her inclusion of groupwork, getting students to talk with each other, and making students explain their thinking in her post-PERC classes. While Hillary made some pedagogical changes based upon her experiences, she did not embrace the multiple roles and identity transformation required of a successful PERC teacher and exited the program.

### Biggest transformations

#### Becoming believers

While some teachers already possessed or readily adopted identities in line with being a PERC teacher and one did not change, a third group of teachers experienced dramatic identity transformations when implementing the model and achieved success in the PERC Program, executing the model in a way that demonstrated the PERC target behaviors and developing strong mentoring relationships with their TAS. The biggest transformations happened for teachers who entered the PERC Program with lecturing as their preferred mode of instruction. As students, they had been successful learners in that class format and felt competent as lecturers themselves. They loved their content and truly enjoyed explaining it to others. This is why they entered teaching, and it is where they got their greatest professional satisfaction. Teachers with content expert identities genuinely did want students to learn, frequently explaining concepts over and over in an effort to impart the content. Lily, mentoring a reluctant novice PERC teacher during the Summer Institute, shared that one of her own PERC mentors initially had a hard time believing that students could learn anything that did not first come out of her own mouth. This experienced PERC teacher’s transformation gave Lily insight into typical teacher struggles, making her an empathetic and effective mentor herself. For teachers such as Lily’s mentor, their primary identity in the classroom was content expert, and their role was of explainer. Yet, when these content-expert teachers seriously examined the outcomes of this traditional classroom structure for their students—from daily engagement levels to high stakes test performance—they acknowledged that something was not working. Content-expert teachers usually attributed the lack of success in their traditional classrooms to the students, claiming that they were different from the students in suburban schools with whom they themselves had been educated. A principal almost bragged, “Our students have very low tolerance for mediocre teaching.” Thus, these educators realized that it was not that the teachers needed to become more effective lecturers. Instead, the students required a completely different, student-centered pedagogy, one embodied by the PERC Program that involved different roles for teachers as well as their students.

For some content-expert teachers, epiphanies happened quickly. In cases such as Andrew, the first visit to a PERC class yielded a dramatic response, “Within the first 15 minutes I knew that this is what our school needed.” For others, their first summer immersion in the student-centered PERC classroom, surrounded by student success and dominated by TAS leadership, made skeptical teachers into believers. Such teachers commented on the fact that all the students in the PERC classroom were engaged and claimed that they believed that more learning was happening than in other classrooms they had observed. These initially uncertain teachers were committed to student success and ultimately believed that the PERC Program would facilitate that within their schools. They had needed to see it in action in order to believe that peer-led learning could be effective with students in urban classes, but seeing was indeed believing for them.

#### Learning to implement

While most participating teachers claimed that their induction into the PERC Program convinced them that the model would be effective for their students, that did not mean that the ultimate transformation in identity and practice was easy. Some teachers described feeling like a “novice,” a term the program adopted for PERC teachers during their first summer in order to prepare teachers for that experience. Some participating teachers articulated a significant amount of struggle with learning to trust their TAS, like Alan admitting, “it is hard to let go the first couple of years.” As Andrew shared,

I think the difficulty is the relinquishing of responsibility, sometimes I find myself wanting to say more. And it’s, although I have got better as the year has progressed this year, being able to shut my mouth, but sometimes I feel like an over bearing parent, “No No No don’t do it that way” instead of what you are supposed to do, let the students make mistakes and learn from that.

Andrew describes this group’s common struggle with transitioning from an identity as content expert to an identity as learning manager.

Some participating teachers initially seemed to believe that the TAS had taken over their role in the classroom, making them feel superfluous or redundant. During PERC class observations, such teachers appeared lost in the classroom during groupwork components of the lesson. Observation records for their lessons include a great deal of evidence of TAS success during groupwork but almost no comments about teacher actions during this major lesson component. Teachers who struggled in this area had relinquished their previous role of content specialist but had not yet adopted a teaching team manager identity. In response to such observations and expressed concerns, coaches worked with these struggling PERC teachers to be more active in the classroom. The coaches modeled the PERC teacher roles and provided explicit guidance and encouragement, both during class and in coaching sessions. Teachers who embraced this coaching made dramatic progress in their transformations, taking on new roles in the classroom that developed their identities in relation to teaching the TAS. For example, just over halfway through his first year in the program, Andrew positively glowed as he explained, “I’ve just had one of those days that makes life worthwhile.” That day he had allowed his TAS to take over the PERC class completely, from starter problem to exit slip. During the PERC lesson, Andrew assessed the TAS using the district’s teacher evaluation framework (Danielson 2014), giving them feedback during TAS class and asking them to write reflections about their own performance. Having abandoned his identity as repository of content knowledge, he reveled in his new identity as mentor of his TAS. Further, Andrew shared that his TAS whom he had taught previously were surprised about his different demeanor in the PERC classroom, indicating that he was not as hard on his students this year. He clarified that, “It’s more of a pastoral learning environment than bark-bark-bark,” explaining to his TAS that he relied upon them to play the role of taskmaster in their groups. Similarly, Alan had to learn that he had an important role to play in the PERC classroom as the TAS were leading content exploration—that of assessing student understanding. Once Alan shifted his identity, he claimed that he was doing a lot more listening and assessing. He believed that this new role ultimately had more impact on student learning because in his former role, he was largely ignorant of what the students had actually learned. Teachers like Alan found a new way to utilize their content expertise through their relationships with their TAS. They shifted from believing that the content is the most important thing in the room to believing that the students were the priority. While such participants reported struggling with learning to implement the model, they tended to seek ways to improve their own effectiveness with being a PERC teacher. They adopted an identity as a learner within the PERC Program.

#### Mentoring TAS

PERC teachers also discussed the new roles that were involved in developing their TAS from marginally successful students to academic leaders. The entire PERC community recognized the challenges of accepting this new role and the shifts required to develop the identity of TAS mentor. As one PERC coach explained, “It’s not just show up and put on the t-shirt and you’re an amazing TAS. There’s a development involved.” One principal argued that effective PERC teachers needed to be analytical thinkers who understand how students learn so that they can teach that to their TAS. When asked what had been difficult about becoming a PERC teacher, Matthew admitted that while the structure of the PERC class was easy for him to implement:

I have struggled through different points of the year with the maturity development of the TAS and their responsibility. Teaching them to be focused and to not fool around with each other has been something that has come up a couple of times and that we worked through, and that’s part of their maturity and development as a person.

However, Matthew embraced this mentoring role and wanted to continue working with his TAS during the summer to ensure their successful progress through the curriculum. These participating teachers appreciated the challenge of developing this new identity of supporting the development of the TAS, acting as their mentor and not just their content instructor.

## Discussion

This study enriches and enhances prior research about STEM teachers’ identities in student-centered classrooms. The language PERC teachers used as they described themselves, their roles and identities, reflects the student-centered rather than teacher-centered structure of the PERC classroom. Their descriptions match the roles the literature ascribes to STEM teachers in student-centered classes (Gordon et al. 2001; Juntunen and Aksela 2013; Kazempour 2009; Moustafa et al. 2013; Tamim and Grant 2013; Yukhymenko et al. 2014) reinforcing what teachers, administrators, teacher educators, and professional developers should expect and prepare for as STEM teachers transition to student-centered instruction. Through their experiences in the PERC Program, participating teachers took on identities much more complex than the ones they had previously embraced, which had focused on being STEM content experts. Whereas in their previous classroom identities involved a single focus on content and delivering it to a single entity of the class, their new identities required them to focus on the learning of each individual student in their classroom and how to be an appropriate teacher for this multiplicity of learners. The difficult-transition teachers who struggled with their concern over content were similar to STEM teachers in the literature who worry about having time to cover the curriculum (Boddy et al. 2003; Kazempour 2009; Keys and Bryan 2001; Tamim and Grant 2013) or student exam performance (Kazempour 2009; Keys and Bryan 2001; Qhobela, 2012; Tamim and Grant 2013) in student-centered classes. Yet, all but one teacher in this study were able to make the transition. They learned to prioritize the identity of assessor described in student-centered literature (Kazempour 2009; Yukhymenko et al. 2014) rather than content dispenser typical of traditional, teacher-centered STEM classrooms. Participating teachers described their identities as shifting away from being the focus of the classroom towards a more supporting role, transitioning from being the instructional star to being the director of learning. These insights are consistent with and further illuminate previous work in this field.

While many of the participating PERC teachers’ experiences were compatible with the literature, some of the teachers’ claims suggested that the PERC model addressed implementation concerns of teachers in other student-centered STEM programs. For example, participating PERC teachers argued that classroom management issues disappeared in PERC classes because TAS kept students engaged, preventing both frustration and boredom within their groups. This belief that PERC improved student behavior contrasted with prior studies of teachers in student-centered programs who worried that losing the teacher-centered structure worsened student behavior (Tamim and Grant 2013). Working in classes where every student was engaged was the favorite aspect of being a PERC teacher for many participants. Additionally, PERC teachers’ belief that PERC increases the amount and complexity of content addressed in classes contrasts to studies where teachers believed that student-centered instruction poses challenges for curriculum coverage (Boddy et al. 2003; Kazempour 2009; Keys and Bryan 2001; Tamim and Grant 2013).

The current study consisted of two case schools involving 13 STEM teachers. While this enabled patterns to emerge across teachers and sites, it limited the depth of exploration of any single teachers’ experience. A case study of an individual teacher would allow detailed analysis of the trajectory of identity transformation that was not possible while working with 13 teachers. The study was set in two schools implementing a specific student-centered instructional model. Further, the study involved STEM teachers in STEM classes, which limits generalizability to teachers in other subject areas. It is impossible to determine how much of the impacts that the teachers describe are attributable to specific factors of the PERC model and what might be generalizable to other student-centered approaches. The two case schools were both urban schools serving students with limited past academic achievement and coming from backgrounds underrepresented in STEM. Further research would be needed to determine whether teachers in more affluent schools, or suburban or rural schools, or those serving students with prior academic success would experience the same types of transformations. The current case studies identify issues worth pursuing in other contexts and with other programs.

Current PD programs tend to focus on what teachers need to do in their classrooms while neglecting the affective impacts that changing pedagogies might have on the participating teachers. As the current study demonstrates, the ways that new pedagogies affect teachers’ identities and the match between teachers’ existing identities and those required by the new pedagogies strongly affect teachers’ ability to adopt and adapt to student-centered instruction. Further research is needed to help teachers and administrators make appropriate choices about PD that supports identity transitions. The PERC Program has begun to use the insights from this study to shape teacher selection and PD experiences for novice PERC teachers. Early discussion of the PERC Program with prospective administrators and teachers now includes transparency about the challenges as well as benefits of becoming a PERC teacher and participating in a student-centered classroom. The PERC Summer Institute has added specific experiences designed to facilitate teachers’ transition from focusing on content to focusing on students and supporting TAS. Research is being conducted on these experiences to determine their impacts on teachers who enter the program with varying teacher identities.

## Conclusions

This study contributes to the discourse about the complex interactions between STEM teachers’ experiences and their professional identities. It demonstrates that radically changing the learning environment can affect teachers’ identities and their approaches to teaching. Teachers who entered the PERC Program predisposed to identifying themselves as coaches or facilitators experienced minimal stress as they learned to develop and mentor their TAS. Teachers who saw themselves as content deliverers experienced a more radical shift in the ways that they thought about themselves in the classroom after joining PERC. Teachers who made the identity transformation learned to value different experiences in the classroom, redefining teaching to include more roles. These identity changes led the teachers to gain insights about the individual learners in their classroom, seeing the adolescents’ true potential as learners and peer leaders. These changes in the PERC teachers’ identities had positive impacts on their students.

PERC teachers’ identity development involved interactions with peers and administrators, students and coaches. In the PERC Program, TAS played a central role in the teachers’ community of practice. As the participants crafted a new type of teacher-student dynamic (Keiler and Robbins In Press), teachers expanded their identities from teachers of STEM to developers of human potential. Particularly for teachers who initially struggled, PERC coaches played a vital role in facilitating the risk taking in the classroom that led to identity changes. When the teachers trusted their TAS and delegated responsibility to them, they were rewarded with engaged students and high functioning classrooms that resulted in positive evaluations from administrators. These positive outcomes sustained most teachers through the challenges of implementing new pedagogies and teaching a completely novel course, making the new identity of being a learner worthwhile. The teacher whose identity was too rigidly fixed to allow her to collaborate with her TAS did not truly implement the model and did not have the experiences necessary to facilitate change. For the majority of PERC teachers, the PERC Program’s classroom structure and requirement to focus on the development of the TAS had substantial impacts on the ways that the participants thought about themselves as STEM teachers.

## Figures and Tables

**Fig. 1:**
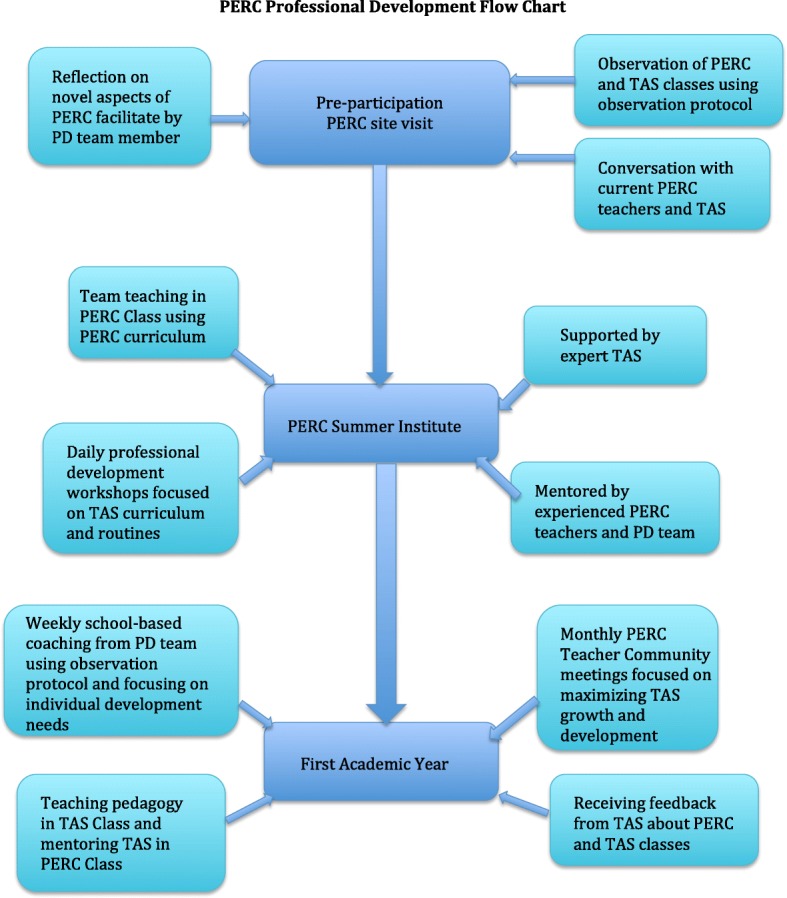
PERC professional development flow chart

**Fig. 2:**
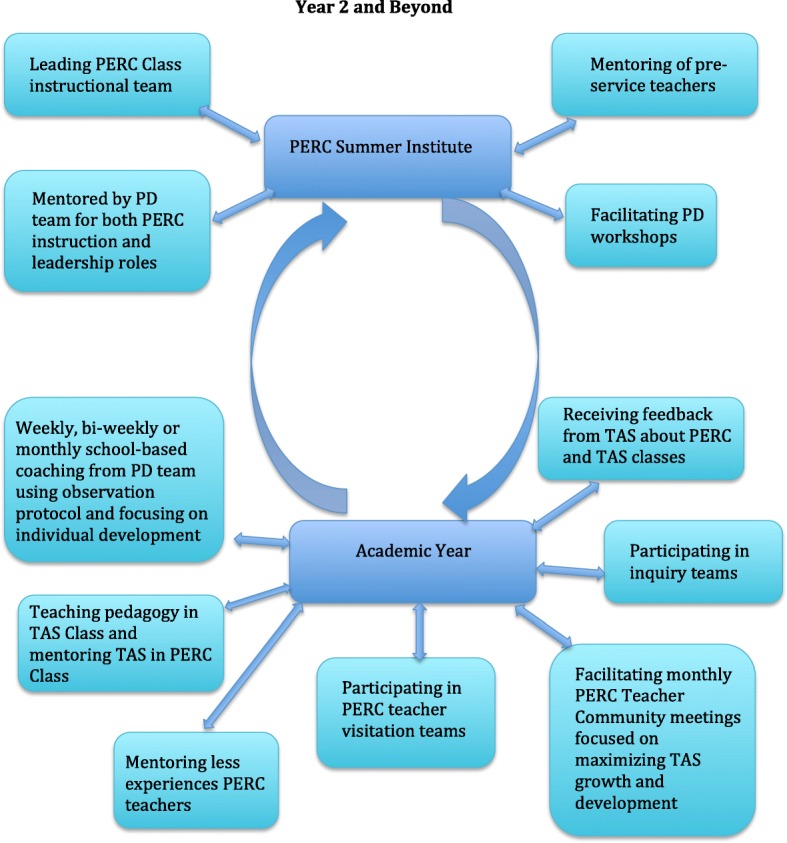
Year 2 and beyond

## Appendix 1

**Table 3:** PERC class target behaviors

Lesson component	Student behaviors	TA scholar behaviors	Teacher behaviors
Do Now (5 min)	• Enter class on time • Sit in TAS group • Show homework to TAS • Complete Do Now task • Ask TAS and other students questions if needed to complete Do Now	• Enter class in time to set up Do Now materials • Pick up TAS folder with record sheets and materials for the lesson • Greet students as they join group • Record student attendance and homework completion • Encourage students to work on Do Now • Ask scaffolding questions to facilitate Do Now completion	• Greet students and TAS as they enter • Encourage students to join groups and start Do Now • Set up materials for lesson • Speak with students needing individual interventions
Lesson introduction/lecture/class discussion (10 min)	• Take notes • Ask teacher or TAS questions when confused or to deepen understanding • Answer teacher’s questions • Participate in group discussions of questions or problems set by the teacher	• Model note-taking for students • Lead group discussions of questions posed or problems set by the teacher • Respond to student questions and answers with scaffolding questions • If explanation is required, break concepts into manageable chunks • Support ELL students in their home language (bilingual TAS)	• Establish motivation for lesson • Introduce content, concepts, and/or skills of an appropriate quantity and complexity • Pose questions for TAS groups to discuss/problems to explore • Facilitate sharing of group discussions with whole class • Transition effectively between group and whole class work
Group work (25 min)	• Work on task assigned by teacher/TAS • Ask TAS or other students questions when confused or to deepen understanding • Collaborate with other students in completing tasks and developing understanding	• Ask scaffolding questions to facilitate task completion and assess understanding • Respond to student questions and answers with scaffolding questions • If explanation is required, break concepts into manageable chunks • Use own completed work as a reference for supporting students • Encourage students to assist each other productively • Give students appropriate positive feedback • Ask teacher and other TAS for support when needed • Support ELL students in their home language (bilingual TAS)	• Listen to TAS group discussion to assess student understanding and TAS effectiveness • Listen to several exchanges within a group before contributing a question or comment • Model effective questioning and positive feedback • Provide whole class intervention if common misconception is identified across groups • Work with individual students or groups who need additional support
Lesson closure (5 min)	• Complete exit slip • Share out group’s ideas/products/answers from day’s lesson • Respond to teacher questions • Ask questions when confused or to deepen understanding • Set a goal • Record homework assignments	• Observe student work on exit slip • Record student daily progress in journal and/or on record sheet • Collect student work • Encourage students to share out and answer teacher’s questions • Ensure that students write down homework	• Assign exit slip • Observe student progress on exit slip • Ask questions to assess learning from lesson • Prepare students for next lesson • Ask students to set a goal • Assign homework

The following chart outlines behaviors that occur during different lesson components included in typical PERC classes. An individual lesson might include some or all of these components

## References

[CR1] Basu SJ, Barton AC (2010). A researcher-student-teacher model for democratic science pedagogy: connections to community, shared authority, and critical science agency. Equity & Excellence in Education.

[CR2] Basu SJ, Barton AC, Clairmon N, Locke D (2009). Developing a framework for critical science agency through case study in a conceptual physics context. Cultural Studies of Science Education.

[CR3] Beijaard D, Meijer PC, Verloop N (2004). Reconsidering research on teachers’ professional identities. Teaching and Teacher Education.

[CR4] Boddy N, Watson K, Aubusson P (2003). A trial of the five Es: a referent model for constructivist teaching and learning. Research in Science Teaching.

[CR5] Bonner SM, Keiler LS (2015). The effect of peer-facilitated learning on teen leaders.

[CR6] Bonner SM, Somers JA, Rivera GJ, Keiler LS (2017). Effects of student-facilitated learning on instructional facilitators. Instructional Sciene.

[CR7] Bonner SM, Thomas AS (2017). The effect of instructional facilitation on student college readiness. Instructional Science.

[CR8] Burris S, Garton BL (2007). Effect if instructional strategy on critical thinking and content knowledge: using problem-based learning in the secondary classroom. Journal of Agricultural Education.

[CR9] Cates JT, Schaefle SE (2011). The relationship between a college preparation program and at-risk students’ college readiness. Journal of Latinos and Education.

[CR10] Center for Advanced Study in Education (CASE) (2015). Year IV evaluation report: MSPinNYC2.

[CR11] Cohen JL (2008). ‘That’s not treating you as a professional’: teachers constructing complex professional identities through talk. Teachers and Teaching: Theory and Practice.

[CR12] Conley DT (2008). Rethinking college readiness. New Directions for Higher Education.

[CR13] Creswell J (2002). Educational research: planning, conducting, and evaluating quantitative and qualitative research.

[CR14] Creswell JW (2013). Qualitative inquiry and research design: choosing among five approaches.

[CR15] Danielson, C. (2014). *The framework for teaching: Evaluation Instrument, 2013 edition.* www.danielsongroup.org. Accessed 5 Feb 2015.

[CR16] Darling-Hammond L, Richardson N (2009). Research review/teacher learning: what matters?. Educational Leadership.

[CR17] Davis EA, Petish D, Smithey J (2006). Challenges new science teachers face. Review of Educational Research.

[CR18] Dignath C, Büttner G (2008). Components of fostering self-regulated learning among students. A meta-analysis on intervention studies at primary and secondary school level. Metacognition & Learning.

[CR19] Eick CJ, Reed CJ (2002). What makes an inquiry-oriented science teacher? The influence of learning histories on student teacher role identity and practice. Science Teacher Education.

[CR20] Ermeling, B.A. & Gallimore, R. (2014/2015). Close-to-practice learning. Online version of *Educational Leadership, 72*(4). http://www.ascd.org/publications/educational_leadership/dec14/vol72/num04/Close-to-Practice_Learning.aspx.

[CR21] Freedman SW, Applement D (2008). “What else would I be doing?”: teacher identity and teacher retention in urban schools. Teacher Education Quarterly.

[CR22] Gerena L, Keiler L (2012). Effective intervention with urban secondary English language learners: how peer instructors support learning. Bilingual Research Journal.

[CR23] Ginsburg-Block M, Rohrbeck CA, Fantuzzo JW (2006). A meta-analytic review of social, self-concept, and behavioral outcomes of peer-assisted learning. Journal of Educational Psychology.

[CR24] Gordon PR, Roger AM, Comfort M, McGee BP (2001). A taste of problem-based learning increases achievement of urban minority middle-school students. Educational Horizons.

[CR25] Greene JA, Azevedo R (2007). A theoretical review of Winne and Hadwin’s model of self-regulated learning: new perspectives and directions. Review of Educational Research.

[CR26] Grier JM, Johnston CC (2009). An inquiry into the development of teacher identities in STEM career changers. Journal of Science Teacher Education.

[CR27] Gusky TR (2000). Evaluating professional development.

[CR28] Gusky TR, Yoon KS (2009). What works in professional development?. Phi Delta Kappan.

[CR29] Gutstein E (2007). “An that’s just how it starts”: teaching mathematics and developing student agency. Teachers College Record.

[CR30] Huberman A, Miles M, Denzin N, Lincoln Y (1994). Data management and analysis. Handbook of qualitative research.

[CR31] Juntunen M, Aksela M (2013). Life-cycle analysis and inquiry-based learning in chemistry teaching. Science Education International.

[CR32] Kazempour M (2009). Impact of inquiry-based professional development on core conceptions and teaching practices: a case study. Science Education.

[CR33] Keiler L (2011). Am I still the teacher? The teachers’ roles in a peer-mediated learning environment.

[CR34] Keiler L (2011). An effective urban summer school: students’ perspectives on their success. The Urban Review.

[CR35] Keiler, L. S., & Robbins, K. (In Press). Roles, responsibilities, and relationships in student-centered STEM classes. In *STEM teaching and learning in the 21st century: meeting the global challenges of standards, equity, evaluation and change*. New York: Peter Lang.

[CR36] Keys CW, Bryan LA (2001). Co-constructing inquiry-based science with teachers: essential research for lasting reform. Journal of Research in Science Teaching.

[CR37] Kistner S, Rakoczy K, Otto B, Dignath-van Ewijk C, Büttner G, Klieme E (2010). Promotion of self-regulated learning in classrooms: investigating frequency, quality, and consequences for student performance. Metacognition & Learning.

[CR38] Komives SR, Mainella FC, Longerbeam SD, Osteen L, Owen JE (2006). A leadership identity development model: applications from a grounded theory. Journal of College Student Development.

[CR39] Krueger RA, Casey M (2009). Focus groups: a practical guide for applied research.

[CR40] Kvale, S. (1996). *Interviews: An introduction to qualitative research interviewing*. London: Sage.

[CR41] Lew YL (2010). The use of constructivist teaching practices by four new secondary school teachers: a comparison of new teachers and experienced constructivist teachers. Science Educator.

[CR42] Lewis GM (2014). Implementing a reform-oriented pedagogy: challenges for novice secondary mathematics teachers. Mathematics Education Research Journal.

[CR43] McDonough PM (1997). Choosing colleges: how social class and schools structure opportunity.

[CR44] McMaster KL, Fuchs D, Fuchs LS (2006). Research on peer-assisted learning strategies: the promise and limitations of peer-mediated instruction. Reading & Writing Quarterly.

[CR45] McMillan JH, Schumacher S (2006). Research in education: evidence-based inquiry.

[CR46] Merriam S (2009). Qualitative research: a guide to design and implementation.

[CR47] Merseth KK, Sommer J, Dickstein S (2008). Bridging worlds: changes in personal and professional identities of pre-service urban teachers. Teacher Education Quarterly.

[CR48] Miles M, Huberman A, Saldaña J (2014). Qualitative data analysis: a methods sourcebook.

[CR49] Morrison M (2004). Risk and responsibility: the potential of peer teaching to address negative leadership. Improving Schools.

[CR50] Moustafa A, Ben-Zvi-Assaraf O, Eshach H (2013). Do junior high school students perceive their learning environment as constructivist?. Journal of Science Education and Technology.

[CR51] National Council for the Teaching of Mathematics. (2000). *Executive summary: principles and standards for school mathematics.* https://www.nctm.org/uploadedFiles/Standards_and_Positions/PSSM_ExecutiveSummary.pdf. Accessed 27 July 2018.

[CR52] NGSS Lead States (2013). Next generation science standards: for states, by states.

[CR53] Odom AL, Bell CV (2015). Associations of middle school student science achievement and attitudes and science with student-reported frequency of teacher lecture demonstrations and student-centered learning. International Journal of Environmental & Science Education.

[CR54] Partnership for 21st Century Skills. (2015). *P12 framework definitions*. New York: http://www.p21.org/storage/documents/docs/P21_Framework_Definitions_New_Logo_2015.pdf. Accessed 27 July 2018.

[CR55] Perels F, Gürtler T, Schmitz B (2005). Training of self-regulatory and problem-solving competence. Learning and Instruction.

[CR56] Proweller A, Mitchener CP (2004). Building teacher identity with urban youth: voices of beginning middle school science teachers in an alternative certification program. Journal of Research in Science Teaching.

[CR57] Qhobela, M. (2012). Using argumentation as a strategy of promoting talking science in a physics classroom: What are some of the challenges? *US-China Education Review*, B 2, 163–172.

[CR58] Rex LA, Nelson MC (2004). How teachers’ professional identities position high-stakes test preparation in their classrooms. Teachers College Record.

[CR59] Robinson DR, Schofield JW, Steers-Wentzell K (2005). Peer and cross-age tutoring in math: outcomes and their design implications. Educational Psychology Review.

[CR60] Roscoe RD, Chi MTH (2008). Tutor learning: the role of explaining and responding to questions. Instructional Science: An International Journal of the Learning Sciences.

[CR61] Schunk Dale H., Ertmer Peggy A. (2000). Self-Regulation and Academic Learning. Handbook of Self-Regulation.

[CR62] Showers B, Joyce B (1996). The evolution of peer coaching. Educational Leadership.

[CR63] Stake RE (2010). Qualitative research: studying how things work.

[CR64] Stanton-Salazar RD, 2001. Manufacturing hope and despair: the school and kin support networks of U.S.-Mexican youth. Sociology of education series. Teachers College Press: Markiston.

[CR65] Tai RH, Lui CQ, Maltese AV, Fan X (2006). Planning for early careers in science. Science.

[CR66] Tamim SR, Grant MM (2013). Definitions and uses: case study of teachers implementing project-based learning. Interdisciplinary Journal of Problem-Based Learning.

[CR67] Thomas AS, Bonner SM, Everson HT, Somers JA (2015). Leveraging the power of peer-led learning: investigating effects on STEM performance in urban high schools. Educational Research and Evaluation.

[CR68] Topping K (2001). Peer assisted learning: a practice guide for teacher.

[CR69] Topping KJ (2005). Trends in peer learning. Educational Psychology.

[CR70] Tschannen-Moran B, Tschannen-Moran M (2011). The coach and the evaluator. Coaching: The New Leadership Skill.

[CR71] van den Berg Rudolf (2002). Teachers’ Meanings Regarding Educational Practice. Review of Educational Research.

[CR72] van Huizen P, van Oers B, Wubbels T (2005). A Vygotskian perspective on teacher education. Journal of Curriculum Studies.

[CR73] Yin RK (2014). Case study research: design and methods.

[CR74] Yukhymenko MA, Brown SW, Lawless KA, Brodowinska K, Mullin G (2014). Thematic analysis of teacher instructional practices and student responses in middle school classrooms with problem-based learning environment. Global Education Review.

[CR75] Zimmerman BJ (2008). Investigating self-regulation and motivation: historical background, methodological developments, and future prospects. American Educational Research Journal.

